# The use of everolimus in the treatment of neurocognitive problems in tuberous sclerosis (TRON): study protocol for a randomised controlled trial

**DOI:** 10.1186/s13063-016-1446-6

**Published:** 2016-08-11

**Authors:** Elizabeth Randell, Rachel McNamara, D. Mark Davies, Eleri Owen-Jones, Nigel Kirby, Lianna Angel, Cheney Drew, Rebecca Cannings-John, Michelle Smalley, Anurag Saxena, Emer McDermott, Laura Stockwell, Petrus J. de Vries, Kerry Hood, Julian R. Sampson

**Affiliations:** 1 South East Wales Trials Unit, Centre for Trials Research, Cardiff University, 7th floor Neuadd Meirionnydd, Heath Park, Cardiff, CF14 4YS UK; 2 Institute of Cancer & Genetics, Cardiff University School of Medicine, Institute of Medical Genetics Building, Heath Park, Cardiff, CF14 4XN UK; 3 Department of Clinical Neuropsychology, Cardiff and Vale University Health Board (University Hospital of Wales), Heath Park, Cardiff, CF14 4XW UK; 4 School of Psychology, Aras an Phiarsaigh, Trinity College, Dublin 2, County Dublin, Ireland; 5 Division of Child & Adolescent Psychiatry, Department of Psychiatry and Mental Health, University of Cape Town, 46 Sawkins Road, Rondebosch, 7700 South Africa

**Keywords:** Tuberous sclerosis, Neurocognitive, Everolimus, TSC, Epilepsy, Randomised controlled trial

## Abstract

**Background:**

Tuberous sclerosis complex (TSC) is a genetic disorder affecting about 1 in 6000 people and is characterised by the development of tumours in many organs, including the skin and kidneys, and by a range of neurological and neuropsychiatric manifestations. TSC-associated neuropsychiatric disorders (TAND) occur in the majority of those with TSC, and they have a significant impact on patients and their families, given the everyday impact of TAND on education, employment, family and social life. The potential benefits of better treatment for TAND therefore include reduction in health care demands and wider benefits for patients and their carers.

**Methods/design:**

We have planned a single-centre, two-arm, individually randomised, phase II, double-blind, placebo-controlled trial of everolimus versus placebo in the treatment of neurocognitive problems in patients with tuberous sclerosis. Everolimus is a licensed medicine in this patient group, but for a different target of effect. The present trial is a proof-of-principle study developed to provide effect size estimates which may be used to inform the design of subsequent trials. Forty-eight patients aged 16–60 years with tuberous sclerosis who have an IQ >60 and a significant deficit (at least −2 SD) in one or more primary outcome measures will be randomly allocated in a ratio of 2:1 to receive everolimus or placebo, respectively. Participants will be assessed for eligibility and then be started on study medication 4 weeks later. They will then be randomised and receive placebo or everolimus for 24 weeks. Neurocognitive and safety assessments will be carried out at baseline and weeks 4, 12, 24 and 36.

**Discussion:**

This study is designed to determine the effect sizes of treatment with everolimus or placebo for 6 months on specific neurocognitive functions—recall memory (verbal and non-verbal) and executive function—in people affected by TSC who have significant deficits in these functions. These data will provide new evidence to determine whether larger-scale trials are indicated and to explore suitable outcome measures and analytical methods for neurocognitive trial design.

**Trial registration:**

ISRCTN09739757. Registered on 28 Dec 2011.

**Electronic supplementary material:**

The online version of this article (doi:10.1186/s13063-016-1446-6) contains supplementary material, which is available to authorized users.

## Background

Tuberous sclerosis complex (TSC) is a rare genetic disorder affecting approximately 1 in 6000 people and is caused by mutations in the *TSC1* or *TSC2* gene. It is characterised by the development of benign tumours in many organs, including the skin, kidneys, heart, eyes, lungs and brain. Brain involvement can lead to seizures, neurocognitive deficits and behavioural and developmental disorders. TSC-associated neuropsychiatric disorders (TAND) occur in the majority of those with TSC, even in the approximately 50 % of patients with normal IQ (>70) [1–3], and they are rated as the most significant disease manifestations by patients and families because of their everyday impact on education, employment, family and social life [4]. Better treatment options for TAND have the potential to both reduce health care demands and offer wider benefits for patients and their carers.

In the past, it was thought that the cognitive, behavioural and neurological manifestations of TSC might be entirely attributable to tubers—characteristic areas of cerebral cortex or sub-cortical white matter—which occur in most people with TSC. However, recent molecular and cellular insights indicate that the intracellular consequences of *TSC1* and *TSC2* gene mutations may also play a direct and potentially reversible role [5]. New imaging techniques have revealed subtle abnormalities of brain microstructure in otherwise normal-appearing white matter [6]. The TSC1 and TSC2 proteins (hamartin and tuberin, respectively) form a complex that acts to suppress mammalian target of rapamycin complex 1 (mTORC1) signalling; thus, disruption of the TSC1-TSC2 complex (as a consequence of mutations in either *TSC1* or *TSC2*) leads to excessive mTORC1 signalling. The effects of this have been demonstrated in immunohistochemical and immunoblotting studies that have shown activation of the downstream effectors of mTORC1, S6 kinase and phospho-S6 in tuberous sclerosis lesions in both pre-clinical models and patients [7]. In pre-clinical trials, treatment with mTORC1 inhibitors shrank or prevented kidney tumours, improved memory and learning, stopped or prevented epilepsy, and improved survival in transgenic *Tsc1*- and *Tsc2*-mutant mice [8–10]. Published findings from the *Tsc2* study [9] came at a time which served to reinforce interest in the neurocognitive trials in humans. Clinical trials using the mTORC1 inhibitors sirolimus and everolimus have demonstrated significant reductions in brain and kidney tumour volume, along with acceptable safety and tolerability, in patients with tuberous sclerosis [11–15]. These findings led to licensing of everolimus for these indications in the United States and Europe.

In the study of Davies et al. [13, 14], we also monitored memory, learning and executive function as secondary endpoints. During the treatment period, patients with tuberous sclerosis showed improvements in visuospatial and verbal recall memory and in executive function, but not in recognition memory tasks. However, because the trial was not a randomised controlled trial and involved very small numbers of patients, we could not distinguish learning effects due to repeat administration (so-called practice effects) of the tests from drug effects.

Up until 14 September 2015, a total of 11 controlled trials of investigational medicinal products investigating mTORC1 inhibitors in patients with tuberous sclerosis were listed at ClinicalTrials.gov. Several are completed or no longer recruiting. Most are investigating tumour-related outcomes. All are using everolimus or sirolimus (rapamycin). Researchers in one current phase II, non-randomised, open-label trial are investigating the efficacy of everolimus in TSC-associated seizures (NCT01070316). A signal-seeking, randomised, placebo-controlled trial of everolimus for neurocognitive problems in young people with TSC has been completed in the United States, but the results are not yet reported (NCT01289912) [3].

The present trial, TRON, will be an investigation of everolimus in relation to neurocognitive function in adult patients with TSC. It will determine effect sizes to provide evidence for whether larger trials for this indication are appropriate. In contrast to the U.S. study (NCT01289912), it will be focused on adult patients who do not have active seizures or who have well-controlled seizures as well as some degree of cognitive impairment. TAND is multi-level with varying cognitive, behavioural and psychiatric manifestations [3]. In order to differentiate between these, the principal level investigated here through the primary outcomes is the ‘neuropsychological’ level, which will be referred to as *neurocognition*.

## Methods/design

### Study design

**Fig. 1:**
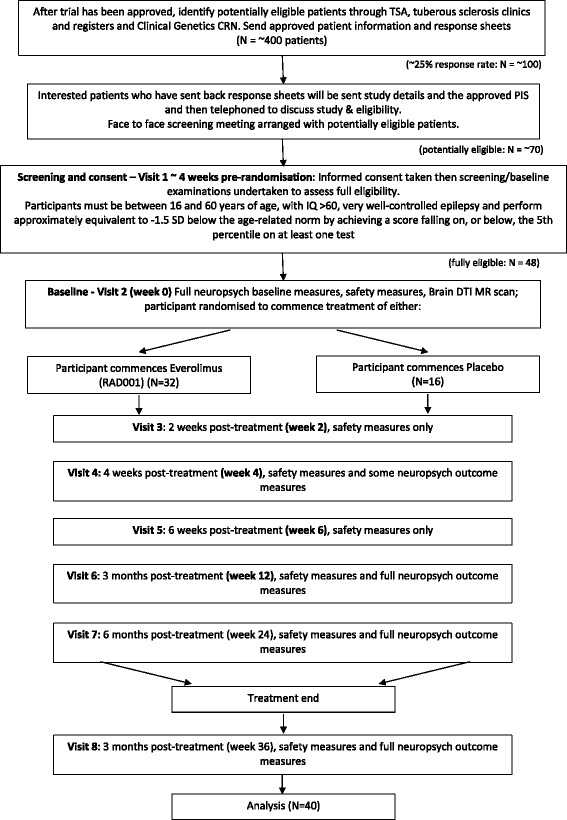
TRON trial design flowchart indicating anticipated patient numbers and dropout. *CRN* Clinical Research Network, *DTI MR* diffusion tensor imaging magnetic resonance imaging, *PIS* patient information sheet, *TSA* Tuberous Sclerosis Association

This is a single-centre, two-arm, individually randomised, phase II, double-blind, placebo-controlled trial of everolimus versus placebo (allocation ratio of 2:1, respectively) in the treatment of neurocognitive problems in patients with TSC. Patients are treated in an outpatient setting at a specialist centre in a large teaching hospital. The present trial is a proof-of-principle study designed to provide effect size estimates which may be used to inform the design of subsequent trials (Fig. 1). Ethical approval was granted by the Wales Research Ethics Committee on 9 November 2011. A Standard Protocol Items: Recommendations for Interventional Trials (SPIRIT) checklist is provided as Additional file 1.

### Research objectives

#### Primary objective

The primary objective is to determine the effect sizes of treatment with everolimus or placebo for 6 months on recall memory and executive function in people with tuberous sclerosis.

#### Secondary objective

The secondary objective is to assess the effects of treatment with everolimus or placebo for 6 months on wider aspects of neurocognitive functioning, seizures and daily life in people with tuberous sclerosis and to assess safety using the National Cancer Institute Common Terminology Criteria for Adverse Events (CTCAE) version 4.0.

#### Exploratory objective

A further exploratory objective is to determine whether an effect of treatment with everolimus or placebo is detectable at 1 month and 3 months after the start of therapy to establish whether any early markers of change are present.

### Sample size determination

The sample size has been calculated on the basis of Fleming’s single-stage procedure [16] and considers the sample size required to assess the change in the experimental group. The control group provides evidence of the learning effect due to repeated assessments. The proposed study seeks to determine effect sizes in relation to the primary outcome measures to enable informed designing of subsequent larger studies. This study is therefore not powered for formal statistical comparison of placebo and study drug groups.

We estimate the learning effect (i.e., the proportion of patients whose memory function improves due to familiarity with the assessments) to be slightly less than 0.15. A proportion of less than 0.15 in the intervention group would therefore indicate that the intervention (everolimus in the treatment of neurocognitive problems in tuberous sclerosis) does not warrant further investigation. A proportion of at least 0.35 would provide sufficient evidence for further investigation of this intervention. Values between these would be discussed in depth by members of the trial team.

Therefore, to test the null hypothesis that the proportion of patients in the intervention group who improve their memory function by 1 SD is at most 0.15 against the alternative hypothesis that the proportion of patients in the intervention group who improve their memory function by 1 SD is at least 0.35, with 80 % power and a one-sided α of 0.05, we require a total sample size of 48 (i.e., 32 who receive the intervention and 16 control subjects). This sample size is inflated to allow for 20 % loss to follow-up.

### Recruitment

Patients aged between 16 and 60 years with tuberous sclerosis will be invited to take part in the trial. The trial will be advertised through the Tuberous Sclerosis Association (TSA) via its website, a regular update e-magazine and a magazine that is mailed to TSA members (approximately 1000 families). Clinical geneticists throughout the United Kingdom will be made aware of the study through the Clinical Genetics Clinical Research Network, and the study has been adopted by National Institute for Health Research via this network. Awareness of the trial will be raised at appropriate conferences. Genetics services will be approached to act as patient identification centres. Patients attending TSA-supported tuberous sclerosis clinics will also be notified of the trial.

### Screening for eligibility

Entry into the study is carried out through a two-stage process. Those who are likely to be eligible will be offered a screening appointment (visit 1), at which fully informed written consent will be obtained. A screening assessment to confirm or refute eligibility will include a full medical history; current medications; physical examination; blood, urine and (for females) pregnancy tests; and neurocognitive tests. Patients must fulfil all of the inclusion criteria and none of the exclusion criteria, as detailed in Table 1.

**Table 1:** TRON study entry criteria

	Inclusion criteria	Exclusion criteria^a^
1	Definite TSC based on current clinical criteria [29]	Prior treatment with an mTOR inhibitor
2	Male or female aged 16–60 years	Investigational agent taken <30 days prior to randomisation
3	IQ >60 as determined by using WASI and able to participate in direct neuropsychological tests	Surgery in last 2 months
4	A score falling on, or below, the 5th percentile (approximately equivalent to −1.5 SD) in one or more of the primary outcome measures	Previous brain neurosurgery, with the exception of sub-ependymal giant cell astrocytoma removal surgery or radiosurgery 5 or more yearsv ago
5	Calculated GFR >60 ml/min/1.73 m^2^, except in case of renal impairment associated with TSC kidney disease, in which event a calculated GFR should be ≥30 ml/min/1.73 m^2^	Significant haematological abnormality (i.e., haemoglobin <8 g/dl, platelets <80,000/mm^3^, absolute neutrophil count <1000/mm^3^)
6	INR ≤1.5 (anti-coagulation permitted if target INR on stable dose of warfarin or LMW heparin for >2 weeks at time of randomisation)	Urine protein/creatinine >0.02 g/mmol, except in case of renal impairment associated with TSC kidney disease, in which case urine protein/creatinine ratio should be >0.1 g/mmol for exclusion
7	Adequate liver function as shown by serum bilirubin ≤1.5 times ULN, ALT and AST ≤2.5 times ULN	Serum creatinine >1.5 times ULN, except in cases of renal impairment associated with TSC complication of kidneys, where serum creatinine should be >300 μmol/L for exclusion
8	If sexually active, negative pregnancy test in females at the time of informed consent, contraception for males and pre-menopausal females in the study	Uncontrolled hyperlipidaemia (fasting cholesterol >300 mg/dl or >7.75 mmol/L and fasting triglycerides >2.5 times ULN, or diabetes with fasting serum glucose >1.5 times ULN)
9	Seizure-free or stable seizures as defined by no change in type of AEDs in 6 months prior to full recruitment and randomisation at baseline; doses of drugs may have been changed in the 6 months prior to recruitment	History of myocardial infarction, angina or stroke related to atherosclerosis, or any other significant cardiac disease, HIV seropositivity, organ transplant, malignancy other than squamous or basal cell skin cancer
10	Hepatitis B surface antigen-negative, hepatitis C antibody-negative	Lymphangioleiomyomatosis with FEV_1_ < 70 % of predicted, or any other restrictive pulmonary disease
11	All patients must be able to communicate well with the investigator, to understand and comply with the requirements of the study, and to understand and sign the written informed consent form	Bleeding diathesis or on oral anti-vitamin K medication other than low-dose warfarin
12	Female patients of childbearing potential must be prepared to use two acceptable methods of contraception (e.g., intra-uterine device plus condom, spermicidal gel plus condom, diaphragm plus condom) from the time of screening	Pregnancy/lactation
13		Live vaccine required during trial
14		Use of strong inhibitor of CYP3A4
15		Use of strong inducer of CYP3A4, except for anti-epileptic drugs
16		Intercurrent infection at time of randomisation
17		Inability to complete study materials (outcome measures) in English
18		History of significant trauma-related cognitive deficit
19		Impairment of gastrointestinal function or gastrointestinal disease that may significantly alter the absorption of everolimus (e.g., pancreatic insufficiency)
20		Known sensitivity to everolimus or other rapamycin analogues or to its excipients
21		Inability to attend scheduled visits

*Abbreviations: AED* anti-epileptic drug; *ALT* alanine aminotransferase, *AST* aspartate aminotransferase, *CYP3AE* cytochrome P450 3AE, *FEV*_*1*_ forced expiratory volume in 1 second, *GFR* glomerular filtration rate, *INR* international normalised ratio, *LMW* low molecular weight, *mTOR* mammalian target of rapamycin, *TSC* tuberous sclerosis complex, *ULN* upper limit of normal, *WASI* Wechsler Abbreviated Scale of Intelligence

^a^Patients meeting any of the exclusion criteria will be excluded from entry into or continuation in the study unless sponsor approval is obtained

### Informed consent

Eligible patients may only be included in the study after providing written (witnessed, where required by law or regulation), informed consent, or, if incapable of doing so, after such consent has been provided by a legally acceptable representative of the patient. Informed consent will also be taken from the individual who accompanies a patient to the assessment visits – whether that is their carer, parent, partner or someone who knows them well – as they will be asked to complete assessments at various time points.

### Randomisation and blinding

Randomisation will be carried out by the South East Wales Trials Unit, using a computer-generated allocation sequence according to a 2:1 for intervention-to-control ratio. The participant’s unique identification number and allocation will be double-blinded so that the participant, clinician, research psychologist and trial statistician will not know to which treatment group the participant has been allocated.

The trial pharmacy will hold details of patients’ treatment allocations and can maintain 24-h cover to unblind in case of an overdose. For all other emergencies, physicians should stop the drug (as unblinding would not affect symptom management) and contact a member of the clinical team as soon as possible afterwards. For non-emergencies, the treating physician should contact a trial clinician to discuss the need to unblind or stop treatment.

### Treatment

The daily investigational drug dose will be 5 mg of everolimus administered for 6 months as two oral 2.5-mg tablets once daily, but with adjustment to achieve trough blood levels of 3–10 ng/ml. A lower dose (as compared with the dose used to treat renal angiomyolipoma [AML]) has been chosen to mitigate side effects, whose frequency and severity may be dose-related. The appearance of placebo medication will be identical to that of active drug to maintain the blinding. Pharmacy staff will be unblinded and will not reveal treatment allocation to anyone without permission from a member of the clinical team or investigational team. Trough blood levels of everolimus will be measured at each study visit and reported to the study doctor through the trials unit. Mock levels for patients on placebo will also be reported in proportion to those for patients on active drug. The study clinicians will then make decisions regarding dosage changes.

Treatment will be dispensed at visits 2, 4 and 6. Patients will take the medication at the study site at visits 3–7 and at home on all other treatment days. Compliance will be monitored by pill counts at each visit. Samples for drug levels (pharmacokinetic assessments) will be taken at visits 3–7 inclusive.

### Safety information and clinical management

Adverse events (AE) are defined as the appearance or worsening of any undesirable sign, symptom or medical condition occurring after starting the study drug or placebo, even if the event is not considered to be related to the investigational medicinal product (IMP). The most common AE are listed in Table 2, and further information on frequency categories of adverse reactions is reported in Additional file 2.

**Table 2:** Common adverse events

Most frequently observed AE resulting from study drug	Most frequently observed laboratory abnormalities
Mouth ulcers	Neutropenia
Rash	Thrombocytopenia
Infections	Hypercholesterolemia
Diarrhoea	Hypertriglyceridemia
Fatigue	Hypophosphatemia
Headache	
Anorexia	
Nausea	
Vomiting	
Non-infectious pneumonitis	

*AE* adverse events

All AE will be collected, recorded and reported in accordance with good clinical practice and the requirements of the Medicines for Human Use (Clinical Trials) Regulations 2004. Their severity will be graded according to the CTCAE. Once detected, they will be treated appropriately and monitored until resolution, unless deemed by the investigator as not necessary and documented accordingly. Treatment may include interruption, modification or discontinuation of study drug treatment and changes in the frequency or nature of assessments, hospitalisations or any other medically required interventions. Recommended treatment of side effects is detailed in Additional file 3.

Known common side effects of the IMP are listed in the summary of product characteristics (SmPC), which is the reference safety information (RSI) for this trial. Any new relevant information between SmPC updates will be issued in the form of investigator notifications. On the basis of the approved RSI for everolimus, expected AE in patients with tuberous sclerosis are seizures and haemorrhage from AML; additional expected AE in females with lymphangioleimyomatosis complicating TSC are pneumothorax, chest infection, chylothorax and haemoptysis.

AE the nature and severity of which are consistent with the information set out in the SmPC for everolimus will be considered expected. Events ongoing at study completion will be followed as clinically indicated. Furthermore, effects of the study drug may be influenced by products that affect cytochrome P450 3A4 and/or P-glycoprotein. These will be avoided wherever possible or used with caution and documented accordingly.

### Pregnancy

Eligible participants are advised that it is not recommended to become or to make their partners pregnant whilst on, and until 30 days after stopping, the study drug. Pregnancies will be reported and documented as appropriate, and followed to determine outcome, including spontaneous or voluntary termination; details of the birth; and the presence or absence of any birth defects, congenital abnormalities or maternal and/or newborn complications. A pregnancy in the partner of a male participant occurring up to 3 months after stopping the study drug will be reported to the sponsor as soon as possible after consent to report information regarding pregnancy outcome has been obtained from the mother.

### Outcome measures

The neurocognitive measures listed in Table 3 will be used at various study visits (see the neuropsychological assessment schedule in Additional file 4 for full details). Although the test battery at some visits is extensive, patients who have neurocognitive problems will complete only the earliest stages of many tests and will therefore complete their assessments much more quickly than the “maximum times” listed in Table 3. A similar test battery has been used with patients with TSC in a previous study [14].

**Table 3:** Neurocognitive outcome measures

	Name of measure
Eligibility visit screening measures	Wechsler Abbreviated Scale of Intelligence (four subtests) [17]
	Edinburgh Handedness Inventory [18]
Primary outcome measures	Complex Figure test and List Learning test from the BIRT Memory and Information Processing Battery [20]
	Spatial Working Memory and Stockings of Cambridge from the CANTAB [21]
	Telephone search dual task from the Test of Everyday Attention [22]
Secondary outcome measures	Information Processing Battery, spatial span and attentional set-shifting (IDED) from the CANTAB [21]
	Cancellation task and Verbal Fluency from the Controlled Oral Word Association Test [23]
	Symptom Checklist-90-Revised [24]
	Quality of Life in Epilepsy [25]
	Liverpool Seizure Severity Scale [26]
	Vineland Adaptive Behaviour Scales-II (survey form) [27]
	Social Responsiveness Scale – Adult version [28]
	Social Communication Questionnaire [29]
	National Adult Reading Test [19]

*Abbreviations: BIRT* Brain Injury Rehabilitation Trust, *CANTAB* Cambridge Neurocognitive Test Automated Battery, *IDED* intra-dimensional/extra-dimensional

### Study assessments

The timing of assessments required during the study is delineated in the study assessment schedule shown in Additional file 5. Appointments will be postponed and rescheduled for patients who have had a generalised tonic-clonic seizure within 48 h of scheduled neurocognitive testing or a complex partial seizure within 24 h of scheduled neurocognitive testing. At the screening visit, background, demographic and administrative assessments will be carried out which will include relevant medical history/current medical conditions; a physical examination; and blood laboratory examinations as per protocol, including a hepatitis screen and a pregnancy test. Patients will also be assessed for IQ [17], handedness [18] and pre-morbid intellectual function [19]. The clinician will also ascertain eligibility by evaluation based on the inclusion and exclusion criteria of the study.

Following the screening visit (visit 1), eligible patients will be scheduled for the baseline and randomisation visit (visit 2) approximately 4 weeks later. This will be considered week 0, and treatment will commence. As outlined in the study assessment schedule (Additional file 5), patients will then be followed for 6 months, with study visits taking place at week 2 (safety), week 4 (safety, some neurocognition), week 6 (safety), week 12 (full neurocognition) and week 24 (full neurocognition at the end of the treatment phase). Each of these visits will take place within 12 h after the last drug administration. A further assessment will be carried out 12 weeks after the end of the last drug or placebo administration, at week 36. Patients will visit the study site on the required days and may stay overnight if travelling from afar, though no hospitalisation will be required. It is anticipated that regular contact with participants and carers will offer the opportunity to address any issues which may contribute to attrition.

Safety assessments will include physical examinations, vital signs and standard clinical laboratory evaluations of haematology, blood chemistry, urinalysis, AE and serious adverse event (SAE) monitoring at each site visit. Monitoring by telephone will be carried out by the clinical trial team at 1 week after initiation of treatment and each month thereafter during the treatment phase of the study, unless a study visit takes place during that calendar month. See Additional file 5 for a full breakdown of the study assessment schedule.

Efficacy assessments for memory (verbal recall memory and non-verbal recall memory) will be measured using two tests that have been validated in the U.K. population across the age range required for this study and for which multiple parallel forms are available as required for retesting. Attentional/executive function will be measured using two tests from a similarly standardised computerised battery, and another from a further well-validated ‘pen-and-paper’ test battery.

Verbal recall memory will be assessed using the list learning recall task [20]. Non-verbal recall memory will be assessed using the complex figure recall task [20]. Attentional/executive function will be assessed using the Stockings of Cambridge [21] planning task and the Spatial Working Memory test [21], and also using the dual task [22].

Attention will be assessed by using the Rapid Visual Information Processing test, the visual search task, sustained listening tasks [22] and the cancellation task [23]. Executive function will be assessed by using the non-verbal intra-dimensional/extra-dimensional test [21] and the verbal Controlled Oral Word Association Test/FAS test [23]. The spatial span task will assess simple working memory. The Symptom Checklist-90-Revised [24] will be used to assess psychological problems and symptoms of psychopathology and/or psychiatric issues.

The Quality of Life in Epilepsy Inventory [25] will be used to evaluate overall quality of life. Seizure severity will be assessed by using the Liverpool Seizure Severity Scale [26] and a seizure diary. A carer, relative or friend will complete the Vineland Adaptive Behaviour Scales-II [27], the Social Responsiveness Scale – Adult version [28] to assess any autism spectrum symptoms, and the Social Communication Questionnaire [29]. All scheduled safety and outcome assessments will be done at the Clinical Research Facility at the University Hospital of Wales, Cardiff. This facility has a dedicated neurocognitive testing suite.

### Data management and monitoring

Data will be captured on study-specific case report forms (CRFs). CRFs will not constitute source data, and all data entered on the CRFs will be traceable to an original source record (electronic or paper), either as part of the electronic database or in the patient’s file. All data management procedures will be detailed in the trial-specific data management plan.

The sponsor’s monitoring standards will ensure 100 % verification for the presence of informed consent, adherence to the inclusion and exclusion criteria, documentation of SAE, and recording of data that will be used for all primary variables. Additional checks of the consistency of the source data with the CRFs will be performed according to the study-specific monitoring plan. All data will be kept for 15 years, in line with the sponsor’s research governance framework regulations for clinical research. Data will be anonymised and stored confidentially in accordance with the Data Protection Act 1998 on password-protected servers maintained in the Cardiff University network.

A Data Monitoring Committee (DMC) will be established that will consist of an independent chair and two other independent members. The main role of the DMC will be to safeguard the interests of the trial’s participants, review the data periodically and make recommendations to the Study Steering Committee.

### Statistical methods

#### Main analysis

All randomised participants who received at least one dose of study drug will be included in the data analysis. Patients will be analysed according to the treatment they received. We will present data descriptively and determine effect sizes. Results will be presented split by trial arm.

The primary outcome of this study is memory function with improved memory function classed as a 1-SD response on any of the memory tests listed in Table 3. A one-sample chi-square test will be used to determine whether the proportion of patients in the intervention group with improved recall memory at 6 months by 1 SD is at least 0.35. The effect size will be presented alongside a 95 % CI and *p* value. The proportion of patients in the control group displaying improved functioning by 1 SD will be used to highlight the learning effect.

#### Secondary analyses

Secondary outcomes will be presented descriptively at baseline and 6 months, and effect sizes will be determined. The effect (between baseline and 6 months) of the control and intervention arms on wider aspects of neurocognitive function, seizures and daily life will be similarly assessed. Secondary analyses will consider both the primary and secondary outcome measures as dimensions of memory function as continuous variables rather than just the proportion of improved patients over time. In further modelling, we will use repeated-measures multivariate analysis of variance to model patterns of functioning over time at baseline and at 3 and 6 months. In these analyses, we will compare the scores from each neurocognitive test at 6 months between the intervention and control arms and will adjust for the balancing factors. The result will be presented as the (adjusted) difference in test outcomes between the intervention and control groups, along with 95 % CI and *p* value. The use of composite measures of these scales will be explored.

## Discussion

This proof-of-principle trial will be the first to determine the effect sizes of treatment with everolimus or placebo for 6 months for recall memory and executive function in adults with tuberous sclerosis. The goal is to provide evidence for whether larger trials for this indication in an adult population are appropriate.

The findings of this study will advance current knowledge in terms of understanding and treatment of tuberous sclerosis. Participants who receive this intervention have the potential for an amelioration of the neurocognitive deficits associated with tuberous sclerosis. Additional potential benefits exist, as mTOR inhibitors have been shown to reduce the size of renal AMLs that are present in most adults with tuberous sclerosis [11, 13] and also to improve the characteristic facial rash (facial angiofibromatosis) [30]. Wider potential benefits to society include reduced demand on carers and on health and social care services, as well as insights into the mechanistic basis for these problems in patients with tuberous sclerosis and potentially other patient groups.

## Trial status

The trial is sponsored by Cardiff University and is currently ongoing and open to recruitment. The manuscript of this report was drafted according to version 9.0 (15 January 2015) of the trial protocol. The protocol was written according to the SPIRIT statement. The final report will be written in accordance with the Consolidated Standards of Reporting Trials (CONSORT) statement.

## Abbreviations

AE, adverse events; AED, anti-epileptic drug; ALT, alanine aminotransferase; AML, angiomyolipoma; AST, aspartate aminotransferase; BIRT, Brain Injury Rehabilitation Trust; CANTAB, Cambridge Neurocognitive Test Automated Battery; CONSORT, Consolidated Standards of Reporting Trials; CRN, Clinical Research Network; CTCAE, Common Terminology Criteria for Adverse Events; CYP3AE, cytochrome P450 3AE; DMC, Data Monitoring Committee; DTI MR, diffusion tensor imaging magnetic resonance imaging; FEV_1_, forced expiratory volume in 1 second; GFR, glomerular filtration rate; IDED, intra-dimensional/extra-dimensional; IMP, investigational medicinal product; INR, international normalised ratio; LMW, low molecular weight; mTOR, mammalian target of rapamycin; mTORC1, mammalian target of rapamycin complex 1; PIS, patient information sheet; RSI, reference safety information; SAE, serious adverse event; SmPC, summary of product characteristics; SPIRIT, Standard Protocol Items: Recommendations for Interventional Trials; SRS-A, Social Responsiveness Scale – Adult version; TAND, tuberous sclerosis-associated neuropsychiatric disorders; TSA, Tuberous Sclerosis Association; TSC, tuberous sclerosis complex; ULN, upper limit of normal; WASI, Wechsler Abbreviated Scale of Intelligence

## Additional files

Additional file 1: SPIRIT checklist of key issues addressed in the trial protocol. (PDF 59 kb) Additional file 2: Frequency categories of adverse reactions reported in the pooled analysis considered for the safety pooling. (PDF 76 kb) Additional file 3: The recommended treatment of side effects that may occur when receiving everolimus. (PDF 41 kb) Additional file 4: Neuropsychological assessment schedule of all neuropsychological assessments to be carried out as part of the trial. (PDF 53 kb) Additional file 5: Study assessment schedule of all assessments to be carried out as part of the trial. (PDF 46 kb)
