# Supplementary material for: The use of everolimus in the treatment of neurocognitive problems in tuberous sclerosis (TRON): study protocol for a randomised controlled trial
Source: Trials. 2016 Aug 11;17:398. doi: 10.1186/s13063-016-1446-6 (PMC4981993; doi:10.1186/s13063-016-1446-6)
Supplement: Additional file 2: — Frequency categories of adverse reactions reported in the pooled analysis considered for the safety pooling. (PDF 76 kb) [file 13063_2016_1446_MOESM2_ESM.pdf]

Additional File 2. Frequency categories of adverse reactions reported in the pooled analysis considered for the safety pooling.

Adverse reactions are listed according to MedDRA system organ class and frequency category. Frequency categories are defined using the following convention: very common (≥1/10); common (≥1/100 to <1/10); uncommon (≥1/1,000 to <1/100); rare (≥1/10,000 to <1/1,000); very rare (<1/10,000). Within each frequency grouping, adverse reactions are presented in order of decreasing seriousness.

**Infections and infestations**	
Very common	Infections a,
**Blood and lymphatic system disorders**	
Very common	Anaemia
Common	Thrombocytopenia, neutropenia, leukopenia, lymphopenia

Uncommon	Pancytopenia
Rare	Pure red cell aplasia
**Immune system disorders**	
Uncommon	Hypersensitivity
**Metabolism and nutrition disorders**	
Very common	Decreased appetite, hyperglycaemia, hypercholesterolaemia
Common	Hypertriglyceridaemia, hypophosphataemia, diabetes mellitus, hyperlipidaemia, hypokalaemia, dehydration, hypocalcaemia
**Psychiatric disorders**	
Common	Insomnia
**Nervous system disorders**	
Very common	Dysgeusia, headache
Uncommon	Ageusia
**Eye disorders**	
Common	Eyelid oedema

Uncommon	Conjunctivitis
**Cardiac disorders**	
Uncommon	Congestive cardiac failure
**Vascular disorders**	
Common	Haemorrhage b, hypertension
Uncommon	Flushing, deep vein thrombosis
**Respiratory, thoracic and mediastinal disorders**	
Very common	Pneumonitis c, epistaxis
Common	Cough, dyspnoea
Uncommon	Haemoptysis, pulmonary embolism
Rare	Acute respiratory distress syndrome
**Gastrointestinal disorders**	
Very common	Stomatitis d, diarrhoea, nausea
Common	Vomiting, dry mouth, abdominal pain, mucosal inflammation, oral pain, dyspepsia, dysphagia

**Hepatobiliary disorders**	
Common	Aspartate aminotransferase increased, alanine aminotransferase increased
**Skin and subcutaneous tissue disorders**	
Very common	Rash, pruritus
Common	Dry skin, nail disorders, mild alopecia, acne, erythema, onychoclasis, palmar-plantar erythrodysaesthesia syndrome, skin exfoliation, skin lesion
Rare	Angioedema
**Musculoskeletal and connective tissue disorders**	
Common	Arthralgia
**Renal and urinary disorders**	
Common	Proteinuria, blood creatinine increased, renal failure
Uncommon	Increased daytime urination, acute renal failure
**Reproductive system and breast disorders**	

Common	Menstruation irregular e
Uncommon	Amenorrhoea e
**General disorders and administration site conditions**	
Very common	Fatigue, asthenia, peripheral oedema
Common	Pyrexia
Uncommon	Non-cardiac chest pain
Rare	Impaired wound healing
**Investigations**	
Very common	Weight decreased

a Includes all reactions within the 'infections and infestations' system organ class including (common) pneumonia and (uncommon) herpes zoster, sepsis, and isolated cases of opportunistic infections [e.g. aspergillosis, candidiasis, pneumocystis jirovecii (carinii) pneumonia (PJP, PCP) and hepatitis B (see also section 4.4)]

b Includes different bleeding events not listed individually

c Includes (common) pneumonitis, interstitial lung disease, lung infiltration and (rare) pulmonary alveolar haemorrhage, pulmonary toxicity, and alveolitis

d Includes (very common) stomatitis, (common) aphthous stomatitis, mouth and tongue ulceration and (uncommon) glossodynia, glossitis

e Frequency based upon number of women from 10 to 55 years of age in pooled data
