# Supplementary material for: The use of everolimus in the treatment of neurocognitive problems in tuberous sclerosis (TRON): study protocol for a randomised controlled trial
Source: Trials. 2016 Aug 11;17:398. doi: 10.1186/s13063-016-1446-6 (PMC4981993; doi:10.1186/s13063-016-1446-6)
Supplement: Additional file 3: — The recommended treatment of side effects that may occur when receiving everolimus. (PDF 41 kb) [file 13063_2016_1446_MOESM3_ESM.pdf]

Additional File 3. Recommended treatment of side effects.

Known undesirable effects of study drug	Recommended treatment
Infections	Patients are at an increased risk of infection. If an infection occurs, anti-infectives should be prescribed as clinically appropriate. In cases of clinically significant infection, consideration should be given to withhold study medication until resolution of infection.
Non-infectious pneumonitis	If this develops, consultation with a pulmonologist is recommended, recorded and managed appropriately as outlined in the protocol.
Mouth ulcers / stomatitis / oral mucositis	These are treated using appropriate locally available supportive care.

Hyperlipidaemia / hyperglycaemia	These are treated according to local best clinical practice. GP to be informed of any incidences of hyperlipidaemia
Diarrhoea	If attributed to study drug toxicity, this may be treated with loperamide. Other medications may be used as required.
