# Supplementary material for: The use of everolimus in the treatment of neurocognitive problems in tuberous sclerosis (TRON): study protocol for a randomised controlled trial
Source: Trials. 2016 Aug 11;17:398. doi: 10.1186/s13063-016-1446-6 (PMC4981993; doi:10.1186/s13063-016-1446-6)
Supplement: Additional file 4: — Neuropsychological assessment schedule of all neuropsychological assessments to be carried out as part of the trial. (PDF 53 kb) [file 13063_2016_1446_MOESM4_ESM.pdf]

Additional File 4. Neuropsychological assessment schedule.

**Visit 1**<br>**Eligibility visit**<br>Approx 4 weeks Pre-baseline	**Visit 2**<br>**Baseline & randomisation**<br>Week 0<br>Then start treatment	**Visit 4**<br>**4-week assessment**<br>Week 4<br>After 1 mo of treatment	**Visit 6**<br>**3-month assessment**<br>Week 12<br>After 3 mo of treatment	**Visit 7**<br>**6-month assessment**<br>Week 24<br>After 6 mo of treatment.<br>Treatment then ends.	**Visit 8**<br>**Post-treatment assessment**<br>Week 36<br>3 mo after treatment ended	Max Time to administer each test

Randomisation						
WASI (IQ)				WASI (IQ)		60- 90 mins
(must be >60)						
EHI Handedness test						3 mins
List learning (BMIPB) -recall after distraction	List learning (BMIPB) -recall after distraction	List learning (BMIPB) -recall after distraction	List learning (BMIPB) -recall after distraction	List learning (BMIPB) -recall after distraction	List learning (BMIPB) -recall after distraction	10 -15 mins
Complex Figure (BMIPB) immed recall	Complex Figure (BMIPB) immed recall	Complex Figure (BMIPB) immed recall	Complex Figure (BMIPB) immed recall	Complex Figure (BMIPB) immed recall	Complex Figure (BMIPB) immed recall	10 mins
SOC (CANTAB) - minimum moves	SOC (CANTAB) - minimum moves	SOC (CANTAB) - minimum moves	SOC (CANTAB) - minimum moves	SOC (CANTAB) - minimum moves	SOC (CANTAB) - minimum moves	15 mins

SWM (CANTAB) - ≥4 between errors	SWM (CANTAB) - ≥4 between errors	SWM (CANTAB) - ≥4 between errors	SWM (CANTAB) - ≥4 between errors	SWM (CANTAB) - ≥4 between errors	SWM (CANTAB) - ≥4 between errors	10 mins
Dual task (TEA) - dual task decrement	Dual task (TEA) - dual task decrement	Dual task (TEA) - dual task decrement	Dual task (TEA) - dual task decrement	Dual task (TEA) - dual task decrement	Dual task (TEA) - dual task decrement	10 mins
National Adult Reading Test						< 5 mins
	Rapid Visual Information Processing (CANTAB)		Rapid Visual Information Processing (CANTAB)	Rapid Visual Information Processing (CANTAB)	Rapid Visual Information Processing (CANTAB)	7 mins
	Spatial span (CANTAB)		Spatial span (CANTAB)	Spatial span (CANTAB)	Spatial span (CANTAB)	5 mins

	IDED (CANTAB)		IDED (CANTAB)	IDED (CANTAB)	IDED (CANTAB)	10-15 mins
	Verbal fluency test		Verbal fluency test	Verbal fluency test	Verbal fluency test	3 mins
	Cancellation task		Cancellation task	Cancellation task	Cancellation task	5 mins
	Symptom Checklist-90R	Symptom Checklist-90R	Symptom Checklist-90 R	Symptom Checklist-90 R	Symptom Checklist-90 R	15 mins
	QOLIE		QOLIE	QOLIE	QOLIE	10 mins
	Liverpool SS scale	Liverpool SS scale	Liverpool SS scale	Liverpool SS scale	Liverpool SS scale	
Start seizure diary (at wks -4 to week 0)	Seizure diary	Seizure diary	Seizure diary	Seizure diary	Seizure diary	Filled in at home daily
	VABS -2			VABS-2	VABS-2	Filled in by carer/relative (45 mn)
	Social Responsiveness			Social Responsiveness Scale	Social Responsiveness Scale - Adult	Filled in (at home) by carer/relative

	Scale - Adult			- Adult		(20 mn)
	Social Communication Questionnaire			Social Communication Questionnaire	Social Communication Questionnaire	Filled in (at home) by carer/relative (10 mn)
