# Supplementary material for: The use of everolimus in the treatment of neurocognitive problems in tuberous sclerosis (TRON): study protocol for a randomised controlled trial
Source: Trials. 2016 Aug 11;17:398. doi: 10.1186/s13063-016-1446-6 (PMC4981993; doi:10.1186/s13063-016-1446-6)
Supplement: Additional file 5: — Study assessment schedule of all assessments to be carried out as part of the trial. (PDF 46 kb) [file 13063_2016_1446_MOESM5_ESM.pdf]

Additional File 5. Study assessment schedule

Assessment	Screening visit	Baseline + Start treatment	2 weeks	4 weeks	6 weeks	12 weeks	24 weeks (End Treatment)	36 weeks (Follow up)
Time point (weeks)	-4 to -1	0	2	4	6	12	24	36
Visit no.	1	2	3	4	5	6	7	8
Written Informed Consent	X							
Inclusion/Exclusion Criteria	X							

Assessment	Screening visit	Baseline + Start treatment	2 weeks	4 weeks	6 weeks	12 weeks	24 weeks (End Treatment)	36 weeks (Follow up)
Time point (weeks)	-4 to -1	0	2	4	6	12	24	36
Visit no.	1	2	3	4	5	6	7	8
Medical History/ Current Medical Conditions/ TSC check	X							
Hepatitis screen and HIV history	X							
Serum pregnancy test for female patients / ask males if partner pregnant	X	X	X	X	X	X	X	X
Prior/Current concomitant medications	X	X	X	X	X	X	X	
Randomisation		X						

Assessment	Screening visit	Baseline + Start treatment	2 weeks	4 weeks	6 weeks	12 weeks	24 weeks (End Treatment)	36 weeks (Follow up)
Time point (weeks)	-4 to -1	0	2	4	6	12	24	36
Visit no.	1	2	3	4	5	6	7	8
Drug administration		X*	X*	X*	X*	X*	X*	
Vital signs[a]	X	X	X	X	X	X	X	
Physical exam (including Neurological Exam)	X	X		X		X	X	
Neuropsychological outcome measures	X	X		(X)		X	X	X
Spot urine protein:creatinine ratio	X	X	X	X	X	X	X	X
Haematology[b]	X	X	X	X	X	X	X	

Assessment	Screening visit	Baseline + Start treatment	2 weeks	4 weeks	6 weeks	12 weeks	24 weeks (End Treatment)	36 weeks (Follow up)
Time point (weeks)	-4 to -1	0	2	4	6	12	24	36
Visit no.	1	2	3	4	5	6	7	8
Coagula - tion Studies (PTT/INR)	X					X	X	
Serum Chemistry[c]	X	X	X	X	X	X	X	
Serum Lipid Profile[d]	X	X				X	X	
Dipstick Urinalysis[e]	X	X	X	X	X	X	X	X
Spirometry[f]	X	X	X	X	X	X	X	X
PK Assessments			X	X	X	X	X	
Adverse Events	X	X	X	X	X	X	X	X
Seizure diary	X	X	X	X	X	X	X	X
Brain DTI MR Scan		X					X	

[a] Vital signs: pulse, respiration rate, blood pressure, temperature, height, weight.

[b] Haematology: hemoglobin, hematocrit, platelets, red blood cell count, white blood cell count, absolute and differential including neutrophils, lymphocytes, monocytes, eosinophils, basophils.

[c] Serum chemistry: total LDH, fasting glucose, sodium, magnesium, phosphate, potassium, creatinine, blood urea, albumin, total protein, SGOT (AST), SGPT (ALT), total bilirubin, alkaline phosphatase, uric acid, calcium, CK.

[d] Serum lipid profile: total cholesterol, triglycerides, LDL, HDL.

[e] Urinalysis: pH, protein, glucose, blood, ketones, leucocytes.

[f] FEV1, FVC

* Patients are not to take study drug (or placebo) on day on which blood levels are measured until after blood samples have been taken.
